# Machine learning can improve the development of evidence-based dietary guidelines

**DOI:** 10.1017/S1368980022001392

**Published:** 2022-09

**Authors:** Lisa M Bodnar, Sharon I Kirkpatrick, Ashley I Naimi

**Affiliations:** 1 Department of Epidemiology, School of Public Health, University of Pittsburgh, 5128 Public Health, 130 DeSoto St, Pittsburgh, PA 15261, USA; 2 Department of Obstetrics, Gynecology, and Reproductive Sciences, School of Medicine, University of Pittsburgh, Pittsburgh, PA, USA; 3 School of Public Health and Health Systems, University of Waterloo, Waterloo, ON, Canada; 4 Department of Epidemiology, Rollins School of Public Health, Emory University, Atlanta, GA, USA

The Dietary Guidelines for Americans provide dietary advice for health promotion and disease prevention and serve as the cornerstone to all other US Federal nutrition policy and education efforts^(1)^. Defining what populations should eat to optimise health is challenging, however, owing to the complexity of the diet. A recent report by the National Academies of Science, Engineering, and Medicine^(2)^ recommended approaches to improve the process of establishing the Dietary Guidelines to account for this complexity, including a call for more advanced analytic techniques for analysing existing dietary data. In this commentary, we highlight several analytic challenges in nutrition and how they can be addressed using a machine learning, a set of flexible algorithms and methods to model complex relations in data.

It is well recognised that foods are eaten in complex combinations with potential antagonistic and synergistic interactions that may impact long-term health^(3,4)^. Some research has captured this complexity by testing whole-diet interventions, such as those rich in vegetables, fruits, whole grains, fish, nuts and beans in the DASH (Dietary Approaches to Stop Hypertension) trial^(5–7)^. Whole-diet interventions have suggested that the most relevant exposure is the totality of the diet^(3)^, typically conceptualised as a multidimensional and dynamic construct referred to as ‘dietary patterns’^(8)^. Dietary patterns constitute the consumption of an array of foods and beverages in different amounts and combinations. The 2010 version of the Dietary Guidelines for Americans recognised the importance of dietary patterns^(9)^, and its emphasis has remained in each subsequent edition^(10,11)^.

Unfortunately, high-quality randomised trials of whole-diet interventions are much more difficult to conduct than dietary supplement interventions^(12)^. Challenges include their sheer complexity, identifying suitable comparators, blinding participants and allocators, and numerous complications involving potential non-adherence to the intervention protocols^(13)^. As a result, observational studies of dietary patterns and disease risk form a majority of the evidence base for dietary recommendations^(1)^. Consequently, the validity of inferences drawn from observational data is critical for informing evidence-based dietary guidelines. Researchers in nutritional epidemiology tend to focus on improving validity primarily by addressing problems with dietary measurement error^(14,15)^. Yet, despite urging from nutrition experts^(8)^, less research has focused on reducing other potential biases and problems with interpretability that can arise when using conventional methods for analysing nutritional epidemiologic data.

## Converting complex dietary patterns into quantitative, interpretable summaries is difficult

Nutrition researchers have predominantly used two methods for summarising diet patterns as primary exposures: *a posteriori* (data-driven) methods like cluster or factor analysis and *a priori* (researcher-driven) methods like diet indexes^(8,16,17)^. Cluster analysis seeks to identify groups of people with similar diets. However, clustering algorithms cannot measure how well the algorithm fits the data on the basis of measured outcomes. As a result, there is often a large degree of unrecognised uncertainty with respect to the quality of a given clustering analysis^(18)^. Factor and principle component analyses identify constructs that explain variability according to food groups, but results from such analyses in nutrition are often erroneously interpreted as causal effects^(19)^. Unfortunately, translating the first principle component of a combination dietary factors into a meaningful quantity that can be used to evaluate the causal effect of a diet pattern is not straightforward^(20)^. Further, questions have been raised about the performance of these techniques for identifying actionable information about the effects of diet on health^(20)^.

Diet indexes measure adherence to a set of diet recommendations defined *a priori*. The Healthy Eating Index-2015 (HEI-2015) is used to evaluate adherence to the 2015–2020 Dietary Guidelines for Americans^(21)^ (an updated version of the HEI aligning with the 2020–2025 Dietary Guidelines has not yet been released). Like other diet indexes (e.g. the Alternative Health Eating Index, the Mediterranean Diet Score index, DASH score^(22–24)^), the HEI-2015 score includes many dietary components, and the total is derived by summing the scores for each individual component, which are truncated at a maximum value. Although diet indexes consider multiple aspects of the diet, the richness of dietary data is reduced into a score. Further, the scoring is defined subjectively without regard to the components’ relation to a heath outcome.

Indeed, the last three editions of the Dietary Guidelines for Americans have recommended a dietary pattern with a variety of vegetables, whole fruits, low-fat or fat free dairy, a variety of protein foods and oils, and limited in added sugars, saturated fats, Na and moderate alcohol^(9–11)^. The HEI-2015 scoring reflects the equal emphasis of these elements of a healthy diet recommended in the 2015–2020 Dietary Guidelines, whereby each of the thirteen components is given a maximum score of 10^(21)^. The Guidelines and the HEI imply that each component of the diet has equal importance to health (adequate fruit is as important as adequate dairy). Yet, studies have not provided an empirical basis for the weighting of the Dietary Guidelines or HEI-2015 score. Analytic approaches that generate weights of each nutritional component for reducing risk of adverse health outcomes are essential for ensuring systematic, objective results.

## Methods typically used in diet patterns research do not formally account for synergy in the diet

Results from laboratory studies have shown that foods eaten in combinations have synergistic effects on some markers of disease risk^(25–27)^, but exploring or accounting for synergy on a wide range of health outcomes in nutritional epidemiology has been limited. While the aforementioned methods for summarising dietary patterns attempt to account for the multidimensionality of diet with a focus on dietary patterns rather than specific foods or nutrients, they do not necessarily account for potential synergistic effects among dietary components.

The presence of such heterogeneity among dietary components has at least two important consequences. First, even when such synergy is not of primary scientific interest, models used to quantify the relation between diet and health outcomes must account for this heterogeneity to avoid potential bias due to the misspecification of variables in the model^(28–30)^. Second, the Dietary Guidelines recommend a diet pattern for the general population, with some differences according to age or life stage^(10)^. These population-level guidelines do not account for the potential for complex varying causal effects of diet patterns in the population, as highlighted by the aforementioned National Academies of Science, Engineering, and Medicine report^(2)^. Evaluating heterogeneity (e.g. synergy, interaction or effect modification) in the causal effects of dietary patterns on health outcomes can enable researchers to target interventions to populations with the greatest likelihood to benefit^(31)^. Notably, deepening our understanding of the role that dietary synergy can play in improving health outcomes extends beyond observational studies. The complexity of randomised trials to estimate the effects of dietary interventions, and the plethora of data usually collected in the context of dietary trials, provides tremendous opportunity to advance this aspect of nutrition.

Nevertheless, it is nearly impossible with conventional parametric methods to correctly code statistical interactions among dietary components in a regression model in order to avoid misspecification bias and correctly capture heterogeneity in the population. This is due to the dearth of knowledge of dietary synergy on outcomes of interest and the vast number of possible interactive associations in the diet.

## Machine learning methods can help establish evidence-based dietary guidelines

One approach to address the difficulties faced when converting complex dietary patterns into quantitative, interpretable summaries is unsupervised learning, which uses machine learning algorithms to identify clusters of individuals that share unique dietary patterns. Algorithms include k-means^(32)^, k-medoids^(33)^, hierarchical or density-based clustering algorithms^(34)^, which can be used to evaluate patterns in diet shared across individuals in a sample. While they can sometimes suffer from stability problems^(18)^, these algorithms have the potential to aid nutrition researchers in better understanding how data can be used to define or inform dietary patterns causally related to health outcomes, as well as how these components should be weighted relative to one another.

Additionally, machine learning can mitigate other problems, such as underlying heterogeneity in associations of interest. For instance, researchers may be interested in the confounder adjusted causal effect of a diet pattern on the risk of adverse health outcomes. To quantify this risk contrast, one would typically adjust for several variables, including other dietary components, in a logistic regression or other parametric regression model. Misspecification bias will result if this association varies among people depending on factors like their fruit intake, their intake of added sugars or their smoking status. One machine learning approach to avoid this bias by accounting for heterogeneity is called stacked generalisation^(35)^, which combines several machine learning algorithms into one. These algorithms can include standard regression models (i.e. generalised linear models) along with other algorithms like random forests and gradient boosting, which may better address potential synergies^(36)^. Results from this stacked generalisation can be used to obtain valid statistics for causal inference (*P* values, CI) using advanced analytic techniques^(37)^.

Lastly, when researchers are specifically interested in evaluating the presence and magnitude of synergy among dietary components or population characteristics, several newer machine learning methods can be used to this end. For example, ‘causal forests’ can be used to quantify the extent to which a causal effect of interest (e.g. a diet pattern rich in vegetables on health outcomes^(38)^) differs across a host of other variables^(39)^, even when the exact variables that lead to heterogeneity in the treatment effect are unknown. This machine learning approach uses random forests to find those variables that explain the largest degree of heterogeneity in the treatment effect of interest and can estimate the precise magnitude of these differences.

An important caveat to recognise is that questions about the impact of dietary synergy on health outcomes are fundamentally causal^(40)^. This requires that a set of conditions hold, such as counterfactual consistency, positivity, no interference and exchangeability (i.e. no information bias, selection bias and confounding bias). These conditions are not guaranteed in observational or randomised trial data, whether machine learning methods are used or not.

Understanding the complex synergies in dietary patterns, their impact on health outcomes and how we can use these to create interpretable measures of dietary patterns is critical for informing national nutrition guidance. Machine learning approaches hold substantial promise in enabling researchers better use the multidimensional nature of dietary data without heavy reliance on parametric assumptions. However, nutrition researchers should be aware of the limitations of machine learning methods, which include high bias, high mean squared error and less than nominal CI coverage when appropriate techniques are not employed^(37,41)^. The challenges in implementing these approaches require nutrition scientists to collaborate with a multidisciplinary team of investigators to identify policy-relevant research questions, appropriate datasets for this work and the techniques needed to address challenges when implementing machine learning. Teams should carefully consider applications of machine learning to their work on dietary patterns so as to ensure possible limitations have been mitigated and their implications for the findings fully recognised.
